# Effect of a short-term low fermentable oligiosaccharide, disaccharide, monosaccharide and polyol (FODMAP) diet on exercise-related gastrointestinal symptoms

**DOI:** 10.1186/s12970-019-0268-9

**Published:** 2019-01-15

**Authors:** Melanie Wiffin, Lee Smith, Jose Antonio, James Johnstone, Liam Beasley, Justin Roberts

**Affiliations:** 1 Cambridge Centre for Sport and Exercise Sciences, School of Psychology and Sport Science, Anglia Ruskin University, Cambridge, UK; 2 Department of Health and Human Performance, Nova Southeastern University, Davie, Florida, USA

**Keywords:** FODMAP diet, Gastrointestinal symptoms, Nutrition, Recreational athletes

## Abstract

**Background:**

Research has demonstrated that low fermentable oligiosaccharide, disaccharide, monosaccharide and polyol (FODMAP) diets improve gastrointestinal (GI) symptoms in irritable bowel syndrome sufferers. Exercise-related GI issues are a common cause of underperformance, with current evidence focusing on the use of FODMAP approaches with recreationally competitive or highly trained athletes. However, there is a paucity of research exploring the potential benefit of FODMAP strategies to support healthy, recreational athletes who experience GI  issues during training. This study therefore aimed to assess whether a short-term LOW_FODMAP_ diet improved exercise-related GI symptoms and the perceived ability to exercise in recreational runners.

**Methods:**

Sixteen healthy volunteers were randomly assigned in a crossover design manner to either a LOW_FODMAP_ (16.06 ± 1.79 g·d^− 1^) or HIGH_FODMAP_ (38.65 ± 6.66 g·d^− 1^) diet for 7 days, with a one week washout period followed by a further 7 days on the alternate diet. Participants rated their gastrointestinal symptoms on an adapted version of the Irritable Bowel Syndrome-Severity Scoring System (IBS-SSS) questionnaire before and at the end of each dietary period. Perceived ability to exercise (frequency, intensity and duration) in relation to each dietary period was also rated using a visual analogue scale. Resting blood samples were collected prior to and on completion of each diet to determine plasma intestinal fatty acid binding protein (I-FABP) as a marker of acute GI injury.

**Results:**

Overall IBS-SSS score significantly reduced in the LOW_FODMAP_ condition from 81.1 ± 16.4 to 31.3 ± 9.2 (arbitrary units; *P* = 0.004). Perceived exercise frequency (z = 2.309, *P* = 0.02) and intensity (z = 2.687, *P* = 0.007) was significantly improved following a short-term LOW_FODMAP_ approach compared to HIGH_FODMAP_. No significant differences were reported between dietary conditions for plasma I-FABP (*P* > 0.05).

**Conclusions:**

A short-term LOW_FODMAP_ diet under free-living conditions reduced exercise-related GI symptoms and improved the perceived ability to exercise in otherwise healthy, recreational runners. These findings may be explained by a reduction in indigestible carbohydrates available for fermentation in the gut. The therapeutic benefits of LOW_FODMAP_ diets in recreational and trained athletes during sustained training periods warrants further investigation.

## Introduction

Fermentable oligosaccharides, disaccharides, monosaccharaides and polyols (FODMAPs) are short-chain carbohydrates that are widespread in the diet in foods such as fruits, vegetables, dairy, wheat, grains, legumes, and are commonly added to processed foods to improve palatability. The major types of FODMAPs known to be problematic are fructose, lactose, oligosaccharides and polyols, each of which has a distinct mechanism of action.

Fructose is absorbed in the small intestine by two carrier protein transporters, GLUT2 (in the presence of glucose) and GLUT5, which facilitate fructose diffusion across cell membranes [1, 2]. In some individuals, the limited availability of the GLUT5 transporter results in fructose malabsorption when it is present in excess of glucose [3–7]. Fructose malabsorption is commonly reported both within irritable bowel syndrome (IBS) sufferers (45%) and healthy individuals (34%) [5]. Lactose malabsorption occurs when there is insufficient lactase to break lactose down into its component sugars glucose and galactose [5].

Oligosaccharides are generally poorly absorbed, resulting in the undigested carbohydrates being fermented by gut bacteria [5, 8, 9]. This results in gas production and flatulence in both healthy and hypersensitive individuals which may instigate adverse symptoms [10]. Polyols do not have an associated active transport system and are thought to be absorbed by diffusion [6], which is variable across the intestine and between individuals [11]. If the polyol is too large for diffusion, malabsorption may occur [8], resulting in fermentation or an increased osmotic load leading to fluid retention in the small intestine [12, 13].

Cumulatively, the malabsorption of these short chain carbohydrates as part of a habitual diet may result in increased small intestinal water volume which can affect gut motility [6, 8]. It has previously been established that altered gut motility is associated with symptoms which are analogues to IBS and exercise including nausea, diarrhoea and urge to defecate during exercise [14, 15]. A LOW_FODMAP_ diet has been established as an evidence-based approach to reduce symptoms in approximately 75% of patients diagnosed with IBS [16]. Sixty to 70% of patients report a worsening of IBS symptoms after habitual meals [17], and consequently will eliminate foods that they believe trigger their symptoms [18, 19].

It is possible that hypersensitive individuals are more susceptible to an adverse reaction to a HIGH_FODMAP_ diet a result of the mechanistic changes during exercise [14, 15, 20, 21] which can ultimately impact on training and/or performance. Increasing the intensity and duration of exercise corresponds with slower gastric emptying and potential for structural epithelial damage, tight-junction disruption and transient luminal permeability, as demonstrated through acute elevated levels of plasma intestinal-fatty acid binding protein (I-FABP) [22–24]. It is therefore relevant to consider whether a short-term LOW_FODMAP_ diet impacts on habitual levels of I-FABP, or indeed whether markers of intestinal damage are exacerbated or sustained as a result of a HIGH_FODMAP_ diet.

It has been reported that 30-50% of athletes cite GI issues as one of the most common causes of underperformance in endurance events [15]. This likely explains why athletes may eliminate food groups they believe cause GI distress [25–28], with a gluten free diet becoming one of the most common approaches reported [27]. However, there is little evidence that removal of gluten has any performance benefits for non-coeliac athletes and it has been suggested that gluten may not be a specific trigger of GI symptoms once dietary intake of FODMAPs are reduced [29]. Recent evidence has focused on the use of FODMAP approaches to support recreationally competitive or highly trained athletes. However, there is a paucity of research exploring the potential benefit of FODMAP strategies to support healthy, recreational athletes who experience GI issues during endurance training. Therefore, the purpose of this randomised, crossover trial was to investigate the effect of altering FODMAP intake upon the GI symptoms reported by recreational athletes in free-living conditions during habitual training. It was hypothesised that a short-term LOW_FODMAP_ diet would improve GI symptoms and the perceived ability to exercise.

## Materials/methods

### Study design

This study employed a randomised, crossover design. The study was conducted in accordance with the Declaration of Helsinki, and ethical approval was granted by the Faculty of Science and Technology Ethics Committee, Anglia Ruskin University (Project Number: FST/FREP/15/567). All participants provided written informed consent prior to study inclusion. All monitoring procedures took place in the Cambridge Centre for Sport and Exercise Sciences, Anglia Ruskin University under controlled conditions.

Participants were required to be healthy, recreationally active runners (training a minimum of 3 days per week with at least 3 months habitual experience, and satisfactorily complete a health screen questionaire) and prepared to comply with study requirements. Ineligible participants were those with a known health condition (including persistent non-exercise related GI issues), current injury, or recent viral infection. Participants were required to only eat foods in conjunction with the lists provided for each 7 day period, and be prepared to weigh food and keep a detailed food log. All participants reported  no known or diagnosed gut disorders, were not currently following a LOW_FODMAP_, ketogenic or calorie restricted diet, were not currently taking antibiotics or probiotics, and had no known blood disorders or allergies.

### Participants

An *a priori* power calculation was undertaken based on the primary end point being the difference in IBS–SSS before and after the LOW_FODMAP_ diet. It was estimated that 11 participants were needed per dietary condition to have an 80% power to detect a difference within group of > 1 SD of IBS-SSS score using a paired t-test with a one-sided α of 0.05 based on previous data [6]. Participants (*n* = 19) were recruited through personal contacts with local running clubs. One participant withdrew due to the burden of keeping a weighed food diary and two participants were excluded from the final analysis due to dietary non-compliance. Sixteen participants completed all aspects of the study (10 female, 6 male; age: 44 ± 10 years, height: 1.70 ± 0.78 m, body-mass: 69.2 ± 8.8 kg).

### Eating plans and dietary intake

According to previous research which categorized carbohydrates as low or high FODMAP [30–33], two separate food lists were devised for this study containing either high or low FODMAP foods. To protect the integrity of the study, participants were instructed to follow the two diets (with an explanation that the type of carbohydrate was different) with no specific reference to FODMAPs in pre study information. For the purposes of protocol blinding lists were named A and B, although complete blinding was not feasible. In order to mimic dietary choice that athletes make in free-living conditions participants were free to select foods from the list and were individually advised to match their typical dietary and calorie intake and record via a weekly weighed food diary. Participants were provided with example diaries and individually instructed in diary completion, with emphasis on meal breakdown, portion size/weight and weighing procedure. Dietary analyses were undertaken by the same researcher for standardisation using Nutritics Professional Dietary Analysis software (Nutritics Limited, Dublin).

### Experimental procedures

Participants attended the laboratory prior to and immediately after each dietary period, and were requested to be rested (no exercise) in the 24-hour period prior to all laboratory measures. For all visits, on arrival, participants rested for 5 minutes prior to fasted blood sample collection, and were then required to complete a symptom questionnaire (see below). Participants were randomised using a pseudo-random number generator (www.randomizer.org) to start on either the low or high FODMAP condition for 7 days based upon previously reported research [34]. All participants undertook a one week washout period between conditions (in a similar manner to previously reported research [10]) and were requested to return to their normal eating patterns during this period before undertaking the opposing dietary condition. Prior to starting, and throughout the study, participants were requested to continue their normal training routine.

### Blood sampling and analysis

Upon arrival, a venous whole blood sample was collected from participants by a qualified phlebotomist into duplicate 4 mL K3EDTA vacutainers (Greiner Bio-One GmbH, Kremsmunster, Austria). Samples were centrifuged for 10 minutes at 3000 rpm, with aliquotted plasma pipetted into sterile, non-pyrogenic, polypropylene cyrovials (Fisherbrand, Fisher Scientific, Loughborough, UK) and immediately frozen at − 80 °C for later assessment of I-FABP using an ELISA kit (Hycult Biotechnology, Uden, the Netherlands; analytical measurement range: 47 to 3000 pg·ml^-1^; intra-assay variance: 3.2% at 360 pg·ml^-1^, 5.4% at 557 pg·ml^-1^ and 6.6% at 809 pg·ml^-1^). Reagents were prepared in accordance with the manufactures instructions at room temperature. Duplicate plasma samples were thawed to room temperature (22 °C) and diluted 10-fold using the sample dilution buffer. I-FABP was extracted from the plasma samples by the addition of the following reagents to ELISA kit in the following order: diluted plasma samples; diluted tracer; diluted streptavidin-peroxidase. In between additions the tray was covered with foil, incubated at room temperature (22 °C) for 1 hour before washing. Finally tetramethylbenzidine (TMB) substrate was added to each well and the tray was incubated for 30 minutes at room temperature. The reaction was stopped with the addition of the stop solution and gently mixed. Samples were read on a spectrophotometer at an absorbance of 450 nm (Victir 3 multilabel plate reader, PerkinElmer Inc., Llantrisant, UK) and referenced against a calibration curve (logarithmic scale).

### Gastrointestinal symptom monitoring

Prior to, and following each dietary period participants rated individual GI symptoms (i.e. bloating, abdominal pain, flatulence, belching, nausea, diarrhoea, defecation, urge to defecate and constipation) against a standardised 0–100 visual analogue scale (VAS) questionnaire (arbitrary units (au)), with no interference from the research team. Global IBS symptom severity scores (IBS-SSS) were based on accumulated results. Clinically significant change of symptoms was defined as > 20 au on the VAS scale [35]. Participants were also requested to rate their perception of their ability to exercise over the week in which each dietary period occurred. Having recorded their training, participants rated their exercise intensity, duration and frequency based on a category scale (no change 0, improved 1, worsened 2) in comparison to a typical training week. Following this, food diaries were collected and inspected for accuracy, detail and compliance using a second pass interview approach between the researcher and participant.

### Statistical analysis

Statistical analyses were performed using SPSS (IBM, Version 24.0). Normality of data were verified by the Shapiro-Wilks test. Outliers were identified by inspection of box plots > 1.5 IQR in SPSS. A repeated measures ANOVA was used to compare effects of dietary interventions (i.e. nutritional intake, IBS-SSS, I-FABP) with Bonferroni post-hoc assessment where applicable. Where sphericity was violated a Greenhouse Geisser correction was applied. A dependent samples t-test was carried out to assess relative differences between diets where pertinent. Ability to exercise data was analysed using a Wilcoxon signed rank test. An alpha level of *P* ≤ 0.05 was considered statistically significant for all tests. Data are presented as mean ± SE.

## Results

### Dietary intake

No significant differences in mean caloric intake were reported between dietary conditions, or in comparison to habitual intake (F = 2.921, *P* = 0.07, ηp^2^ = 0.173; Table 1). When normalised for body-mass, mean habitual caloric intake (34.12 ± 2.48 kcal·kg^− 1^·d^− 1^) was comparable with both LOW_FODMAP_ (29.04 ± 1.88 kcal·kg^− 1^·d^− 1^) and HIGH_FODMAP_ (32.53 ± 2.08 kcal·kg^− 1^·d^− 1^) conditions (F = 3.053, *P* = 0.063, ηp^2^ = 0.179). For carbohydrate intake, a significant main effect was observed (F = 7.091, *P* = 0.0003, ηp^2^ = 0.336), with participants reporting consuming less total (Table 1) and relative carbohydrate intake during the LOW_FODMAP_ condition compared with the HIGH_FODMAP_ condition (2.79 ± 0.30 g·kg^− 1^·d^− 1^ and 3.91 ± 0.36 g·kg^− 1^·d^− 1^ respectively, *P* = 0.003).

**Table 1:** Mean dietary intake under habitual and FODMAP conditions

Variable	Category	Habitual	LOW_FODMAP_	HIGH_FODMAP_
Total EI	(kcal·d^− 1^)	2355.86 ± 197.10	1999.23 ± 138.43	2269.14 ± 162.11
	(kcal·kg^− 1^·d^− 1^)	34.12 ± 2.48	29.04 ± 1.88	32.53 ± 2.08
Carbohydrates	(g·d^− 1^)	245.77 ± 19.05	193.53 ± 21.56^*^	272.28 ± 26.23
	(g·kg^− 1^·d^− 1^)	3.55 ± 0.22	2.79 ± 0.30^*^	3.91 ± 0.36
FODMAPs	(g·d^−1^)	28.04 ± 4.33	15.75 ± 1.91^*, a^	38.59 ± 6.48
	(g·kg^− 1^·d^− 1^)	0.42 ± 0.07	0.23 ± 0.03^*, a^	0.56 ± 0.09
Protein	(g·d^−1^)	92.13 ± 7.82	94.34 ± 5.81	94.64 ± 6.54
	(g·kg^−1^·d^− 1^)	1.34 ± 0.10	1.35 ± 0.07	1.36 ± 0.08
Fat	(g·d^−1^)	101.95 ± 12.74	88.48 ± 9.88	83.92 ± 5.82
	(g·kg^− 1^·d^− 1^)	1.48 ± 0.17	1.29 ± 0.15	1.21 ± 0.08

Table 1 outlines mean habitual dietary intake and between dietary conditions with data expressed in total amounts per day. *EI =* energy intake. * denotes significant difference between LOW_FODMAP_ and HIGH_FODMAP_ conditions only (*P* < 0.004). ^a^ denotes significant difference to habitual diet (*P* < 0.045)

Total FODMAP intake (including relative to body-mass) was also statistically different between conditions (F = 10.354, *P* < 0.0001, ηp^2^ = 0.425), with post-hoc analysis demonstrating the expected reduction with LOW_FODMAP_ (15.75 ± 1.91 g·d^− 1^) compared with both HIGH_FODMAP_ (38.59 ± 6.48 g·d^− 1^, *P* = 0.004) and habitual conditions (28.04 ± 4.33 g·d^− 1^, *P* = 0.045). There were no significant differences reported for dietary fat (habitual: 1.48 ± 0.17 g·kg^− 1^·d^− 1^; LOW_FODMAP_ 1.29 ± 0.15 g·kg^− 1^·d^− 1^; HIGH_FODMAP_ 1.21 ± 0.08 g·kg^− 1^·d^− 1^; F = 1.446, *P* = 0.253) or protein intake (habitual: 1.34 ± 0.10 g·kg^− 1^·d^− 1^; LOW_FODMAP_ 1.35 ± 0.70 g·kg^− 1^·d^− 1^; HIGH_FODMAP_ 1.36 ± 0.08 g·kg^− 1^·d^− 1^; F = 0.142, *P* = 0.798) between experimental conditions or in comparison to habitual intake (total or relative to body-mass).

### Gastrointestinal symptom scores (overall)

**Fig. 1:**
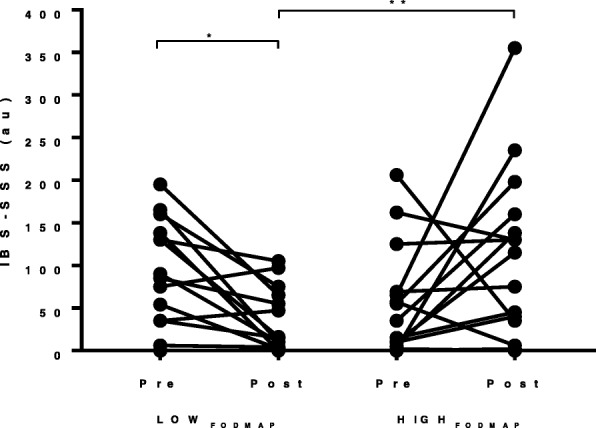
Participant GI symptom scores following each dietary intervention. Displays individual participant accumulated IBS-symptom severity scores following each FODMAP condition. Au = arbitrary units. * denotes significant difference pre-post within group (*P* = 0.004); ** denotes significant difference between dietary conditions (post; *P* = 0.007)

**Fig. 2:**
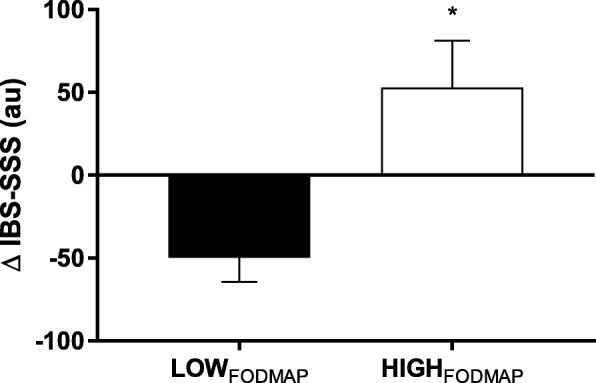
Relative changes in mean GI symptom responses following each dietary condition. The figure demonstrates the mean delta change in GI responses following each FODMAP condition. Au = arbitrary units. * denotes significant difference between conditions (*P* = 0.02)

Mean gastrointestinal symptom scores (IBS-SSS) did not differ between conditions prior to each FODMAP diet (*P* > 0.05), although a wide variance of responses was noted between participants (mean: 66.1 ± 16.3 au; range 0–206 au). A significant diet x time interaction effect was found for IBS-SSS (F = 6.98, *P* = 0.02, ηp^2^ = 0.32), with post-hoc analysis indicating a significant reduction in scores from 81.1 ± 16.4 au (pre) to 31.3 ± 9.2 au (post) with LOW_FODMAP_ (*P* = 0.004; Fig. 1). Although a non-significant increase in IBS-SSS was reported with HIGH_FODMAP_ from 51.1 ± 15.7 au (pre) to 104.0 ± 25.0 au (post; *P* = 0.08); overall end-point scores were significantly different between dietary conditions (*P* = 0.007). Expressed as relative change (Fig. 2), a significant difference was also reported between dietary conditions (mean difference = − 102.7 ± 38.9 au; *t* = − 2.64, *P* = 0.02) in favour of an improvement in responses following a LOW_FODMAP_ approach. Individual responses indicated that 69% of participants (11/16) reported positive effects of the LOW_FODMAP_ diet, in contrast to 25% (4/16) on the HIGH_FODMAP_ diet.

### Gastrointestinal symptom scores (individual)

Table 2 demonstrates mean responses to individual GI symptoms across both dietary conditions. A significant diet x time interaction effect was found for pain (F = 6.861, *P* = 0.019, ηp^2^ = 0.314) with post hoc analyses indicating that end-point scores were significantly different between dietary conditions (4.13 ± 2.52 au for LOW_FODMAP_ and 22.50 ± 6.35 au for HIGH_FODMAP_ respectively, *P* = 0.003), which coincided with significant within-group changes for both LOW_FODMAP_ (*P* = 0.031) and HIGH_FODMAP_ (*P* = 0.028). A significant main effect (time) was reported for flatulence (F = 4.428, *P* = 0.05, ηp^2^ = 0.228), as well as a main effect (diet) for belching (F = 5.686, *P* = 0.03, ηp^2^ = 0.275), although post-hoc analyses were not significant. A significant main effect (diet) was reported for bloating (F = 6.186, *P* = 0.025, ηp^2^ = 0.292), with post-hoc analyses indicating that end-point scores were significantly different between dietary conditions (1.25 ± 0.72 au for LOW_FODMAP_ and 12.69 ± 4.53au for HIGH_FODMAP_ respectively, *P* = 0.021). All symptoms except constipation and defecation recorded a decrease in score on the LOW_FODMAP_, whilst all symptoms except flatulence recorded an increase in score on the HIGH_FODMAP_ diet, however no other significant findings were reported.

**Table 2:** Mean responses to individual GI symptoms across both dietary conditions

	LOW_FODMAP_		HIGH_FODMAP_		
Symptom	PRE	POST	PRE	POST	Interaction
Nausea	4.75 ± 2.86	0.88 ± 0.45	1.56 ± 0.63	4.94 ± 2.22	0.096
Pain	19.75 ± 6.75	4.13 ± 2.52 ^a^	7.63 ± 3.15	22.50 ± 6.35 ^a,b^	0.019*
Belching	3.38 ± 1.92	2.25 ± 1.45	3.56 ± 1.91	7.50 ± 2.99	0.218
Flatulence	14.75 ± 3.66	8.50 ± 2.84	17.38 ± 5.29	13.81 ± 3.57	0.705
Constipation	3.75 ± 2.72	4.00 ± 2.82	0.56 ± 0.34	4.19 ± 2.71	0.369
Diarrhoea	12.19 ± 5.70	3.06 ± 1.83	3.75 ± 3.75	13.13 ± 5.72	0.149
Defecation	2.50 ± 1.88	2.88 ± 2.23	0.37 ± 0.32	8.75 ± 5.21	0.247
Urge to defecate	12.19 ± 4.92	5.13 ± 2.67	7.69 ± 4.96	17.06 ± 6.70	0.155
Bloating	7.88 ± 4.15	1.25 ± 0.72	8.94 ± 4.96	12.69 ± 4.53^b^	0.236

Table 2 displays mean data for individual GI symptoms (arbitrary units) across both dietary interventions. * denotes significant interaction effect (*P* < 0.02). ^a^ denotes significant change within-group (*P* < 0.03). ^b^ denotes significant post-diet difference in comparison to LOW_FODMAP_ (*P* < 0.02)

### FODMAP intake and self-reported ability to exercise

There was a statistically significant median difference in the perceived exercise frequency (z = 2.309, *P* = 0.02) and intensity (z = 2.687, *P* = 0.007) between a LOW_FODMAP_ and HIGH_FODMAP_ diet. No significant median difference was reported in perceived exercise duration on a LOW_FODMAP_ and HIGH_FODMAP_ diet (z = 1.414, *P* = 0.157). Participants were more likely to report that the ability to exercise improved on a LOW_FODMAP_ diet (frequency (4/16), intensity (6/16)), and deteriorated on a HIGH_FODMAP_ diet (frequency (4/16), intensity (9/16)).

### FODMAP intake and intestinal fatty acid binding protein (I-FABP) levels

**Fig. 3:**
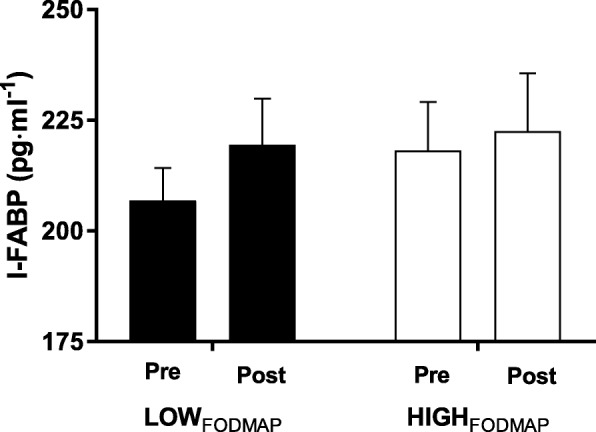
Mean plasma I-FABP concentrations at rest in response to FODMAP conditions. The figure demonstrates the average I-FABP (pg·ml^− 1^) before and after the LOW_FODMAP_ and HIGH_FODMAP_ intervention. No significant differences reported (*P* > 0.05)

The effect of both FODMAP diets on I-FABP levels under resting conditions is shown in Fig. 3. A non-significant increase from 206.93 ± 7.27 pg·ml^− 1^ to 219.46 ± 10.42 pg·ml^− 1^ was noted for LOW_FODMAP_ remaining within expected limits. I-FABP for HIGH_FODMAP_ remained comparable across the intervention (218.21 ± 10.93 pg·ml^− 1^ to 222.60 ± 13.08 pg·ml^− 1^; *P* > 0.05). No significant interaction effects were reported (*P* > 0.05) between dietary conditions.

## Discussion

This study aimed to investigate the perceived effect of acute FODMAP intake on GI symptom severity and ability to exercise in recreational athletes under free-living conditions. Whilst the clinical effectiveness of a LOW_FODMAP_ diet in treating IBS is established in the literature [16, 28], research into the potential therapeutic effects in otherwise healthy, recreational athletes is limited. The main findings from the current study revealed that short-term LOW_FODMAP_ intake significantly improved exercise-related GI symptoms in 69% of participants. These results support recent observations [36] demonstrating reductions in daily GI symptoms in trained athletes on a short-term (6-day) LOW_FODMAP_ diet, as well as reduced GI symptom severity in case studies of a male [37] and female [38] runner. Consistency between these findings infers that both recreational and more trained athletes may benefit from self-prescribed LOW_FODMAP_ approaches in the short-term, providing there is compliance with food choices. This may have implications for longer term FODMAP strategies during sustained training periods, which may provide adjunct nutritional support in maintaining training volume and/or intensity [20] particularly in symptomatic individuals who suffer from GI distress with exercise.

Whilst a LOW_FODMAP_ approach appeared to result in improved scores for most individual symptoms, only responses to perceived pain and bloating were significantly different between conditions following the dietary interventions. This suggests that whilst an improvement in overall GI symptom severity may reflect accumulated reductions in individual symptom responses, the effects of a short-term LOW_FODMAP_ diet may in fact be specific. The reported improvement in perceived pain, in conjunction with reduced experiences of bloating whilst on a LOW_FODMAP_ diet is likely explained by a reduction in intestinal water volume and gas production [6, 10, 39]. Strategies to reduce or minimise such GI symptoms may be important for recreational athletes, especially considering the reported negative impact on exercise training and/ or performance [15, 40, 41]. However, based on the wide inter-individual responses observed across conditions, such findings should be interpreted with caution.

An interesting observation from the current study was the improved perception of exercise frequency and intensity from participants whilst undertaking the LOW_FODMAP_ approach. Although this only reflected perceived changes in the short-term (7 days), this may have implications for sustained approaches where training routines may be disrupted (including volume and intensity) due to GI-related issues. Participants were requested to maintain their typical training routine throughout the study to assess whether perceived changes (in frequency, training duration or intensity) were related to the dietary intervention. Whilst a significant effect was observed for improved perception of exercise frequency and intensity following a LOW_FODMAP_ approach, this only occurred in 25 and 38% of the participants respectively. Only one other study [36] appears to have attempted to standardise training sessions (albeit 2 sessions in a 6-day period) whilst participants undertook an acute LOW_FODMAP_ or HIGH_FODMAP_ diet. In this study, daily GI symptoms for flatulence, urge to defecate and diarrhoea were reportedly improved in the LOW_FODMAP_ condition [36]. However, assessment of participants’ perception of training session ability in relation to the dietary approach was not considered. Further research to establish training related benefits of a LOW_FODMAP_ strategy, particularly with symptomatic individuals, is therefore warranted.

Moderate to high-intensity exercise impacts on gastric emptying, GI transit and intestinal absorption due to GI hypoperfusion and ischemia [15, 22]. Provocation of luminal tight junction proteins (e.g. caludin and occludin) through increased expression of phosphorylation enzymes, reactive oxygen species (ROS) activity and cytokine mediators may lead to acute GI permeability [22, 23, 42] and paracellular transport. Although transient, acute GI disruption may exacerbate nutrient malabsorption, as well as provoke delayed systemic immune responses. Increased residual HIGH_FODMAP_ GI content as a result of malabsorption [43], along with increased delivery of fluid to the colon and reduced GI motility could synergistically impact on perceived severity of symptoms, including acute or transient pain. This may limit the intensity of exercise training, particularly in symptomatic or hypersensitive individuals. The reduction in pain observed in this study, along with improved perception of flatulence and bloating symptoms within-group, indicates that lowering FODMAPs in the diet may support exercise training. Mechanistically, a reduction in fluid re-uptake across the GI epithelia, leading to less fluid and gas build-up pre- or during exercise in response to daily or more habitual LOW_FODMAP_ approaches may assist with sustained exercise tolerance.

In connection with perceived symptom changes, this study also investigated whether a short-term FODMAP approach impacted on basal GI damage via assessment of I-FABP. Whilst it was hypothesised that a HIGH_FODMAP_ diet may lead to an elevated residual level of I-FABP following the short-term intervention, no significant differences were observed within or between conditions. Therefore, even though a HIGH_FODMAP_ approach may have resulted in increased perception of symptom severity, disruption of the epithelial barrier in response to dietary modifications was not evident. Previous research has demonstrated that splanchnic hypoperfusion in response to acute, moderate exercise resulted in elevated I-FABP from 309 ± 46 pg·ml^− 1^ to 615 ± 118 pg·ml^− 1^ in healthy, male volunteers [22], which rapidly returned to baseline concentrations within minutes of recovery. I-FABP is a sensitive marker of small intestinal cell damage. However, rapid changes as observed in the previous study [22] indicate that GI damage is highly transient, and possibly only in response to exercise-based hypoperfusion, which may explain the lack of significant residual findings under resting conditions in the current study. Assessment of I-FABP and/or GI permeability (e.g. urinary lactulose: rhamanose evaluation) in response to daily bouts of exercise in conjunction with a FODMAP approach may, however, provide mechanistic understanding of the potential benefits of a LOW_FODMAP_ diet.

A limitation to the current study observed when implementing a LOW_FODMAP_ diet in free-living conditions was that participants tended to consume fewer calories compared to both habitual and HIGH_FODMAP_ intakes, albeit non-significant. Taking into consideration methodological constraints in maintaining a weighed food diary, this observation was supported by a significant reduction in carbohydrate intake to achieve LOW_FODMAP_ adherence. This finding is consistent with a case study of a female athlete competing in a Multistage Ultramarathon [38], which reported that whilst following a LOW_FODMAP_ approach total daily energy intake did not meet estimated energy requirements. Upon further investigation suboptimal carbohydrate intake rather than protein and fat was observed.

Similar findings have been reported elsewhere [44], in which 29% of participants reported acute weight loss whilst on a LOW_FODMAP_ approach in free-living conditions. Many carbohydrate-rich foods typically consumed by active individuals (e.g. pasta, cereals, bread, energy drinks) are HIGH_FODMAP_, whereas alternative food sources (e.g. rice, corn) may be less palatable or more difficult to substitute. Indeed, in the previous study [44], participants cited that LOW_FODMAP_ approaches were either too complicated, expensive, or did not enjoy the overall taste as reasons for not sustaining the diet. The potentially restrictive or limiting nature of food choices on a LOW_FODMAP_ diet could therefore outweigh GI symptom benefits in the longer term due to weight loss, lethargy, fatigue, perceived effort, cost and/or enjoyment. Furthermore, a sustained energy/carbohydrate reduction in the longer term could also impact on training maintenance and recovery adaptations, and lead to unintended reduced nutrient availability.

However, the finding that acute dietary FODMAP manipulation positively impacted on overall GI symptom severity has pertinent implications for active individuals, particularly those more symptomatic or hypersensitive. Future research should consider whether there is a threshold of symptom severity in the context of exercise above which individuals may benefit from a LOW_FODMAP_ nutritional approach. There is also the need to establish the minimum intervention length required to alleviate GI symptoms in both recreational and trained athletes; as well as how long interventions can be sustained or indeed whether a FODMAP approach can impact on prolonged training periods. This is especially important considering the finding that carbohydrate intake was reduced on the LOW_FODMAP_ diet in free-living conditions, and the known importance of carbohydrates in fuelling regular exercise. Finally, whilst a LOW_FODMAP_ diet is known to significantly affect the gut microflora composition [45–47], the consequences of prolonged LOW_FODMAP_ intake on other types of physical activity, particularly those of a high-intensity or intermittent nature, has yet to be determined.

## Conclusion

In conclusion, this study provides evidence that recreational athletes implementing a short-term LOW_FODMAP_ diet under free-living conditions may experience benefits in exercise-related GI symptoms and perceived improvements in exercise intensity and frequency. However, caution is warranted to minimise unnecessary reductions in total caloric and/or carbohydrate intake that may impact on nutritional quality. Further studies are warranted to investigate the impact of a LOW_FODMAP_ diet on sustained training strategies in healthy, recreationally active individuals and trained athletes.

## Abbreviations

ANOVA: analysis of variance
ELISA: Enzyme-linked immunosorbent assay
FODMAP: Fermentable oligiosaccharide, disaccharide, monosaccharide and polyol diet
GI: Gastrointestinal
IBS: Irritable bowel syndrome
IBS-SSS: Irritable bowel syndrome-symptom severity score
I-FABP: Intestinal fatty acid binding protein
ROS: Reactive oxygen species
